# Stearidonic acid improves eicosapentaenoic acid status: studies in humans and cultured hepatocytes

**DOI:** 10.3389/fnut.2024.1359958

**Published:** 2024-04-04

**Authors:** Ulrike Seidel, Katharina Eberhardt, Michelle Wiebel, Kai Luersen, Ignacio R. Ipharraguerre, Franziska A. Haegele, Peter Winterhalter, Anja Bosy-Westphal, Nils Helge Schebb, Gerald Rimbach

**Affiliations:** ^1^Institute of Human Nutrition and Food Science, University of Kiel, Kiel, Germany; ^2^Institute of Food Chemistry, TU Braunschweig, Braunschweig, Germany; ^3^Chair of Food Chemistry, Faculty of Mathematics and Natural Sciences, University of Wuppertal, Wuppertal, Germany

**Keywords:** ahiflower oil, crossover-study, eicosanoids, HepG2 cells, oxylipins, taurine

## Abstract

**Background:**

Ahiflower oil from the seeds of *Buglossoides arvensis* is rich in α-linolenic acid (ALA) and stearidonic acid (SDA). ALA and SDA are potential precursor fatty acids for the endogenous synthesis of eicosapentaenoic acid (EPA) and docosahexaenoic acid (DHA), which are n3-long chain polyunsaturated fatty acids (n3-LC-PUFAS), in humans. Since taurine, an amino sulfonic acid, is often associated with tissues rich in n3-LC-PUFAS (e.g., in fatty fish, human retina), taurine may play a role in EPA- and DHA-metabolism.

**Objective:**

To examine the capacity of the plant-derived precursor fatty acids (ALA and SDA) and of the potential fatty acid metabolism modulator taurine to increase n3-LC-PUFAS and their respective oxylipins in human plasma and cultivated hepatocytes (HepG2 cells).

**Methods:**

In a monocentric, randomized crossover study 29 healthy male volunteers received three sequential interventions, namely ahiflower oil (9 g/day), taurine (1.5 g/day) and ahiflower oil (9 g/day) + taurine (1.5 g/day) for 20 days. In addition, cultivated HepG2 cells were treated with isolated fatty acids ALA, SDA, EPA, DHA as well as taurine alone or together with SDA.

**Results:**

Oral ahiflower oil intake significantly improved plasma EPA levels (0.2 vs. 0.6% of total fatty acid methyl esters (FAMES)) in humans, whereas DHA levels were unaffected by treatments. EPA-levels in SDA-treated HepG2 cells were 65% higher (5.1 vs. 3.0% of total FAMES) than those in ALA-treated cells. Taurine did not affect fatty acid profiles in human plasma *in vivo* or in HepG2 cells *in vitro*. SDA-rich ahiflower oil and isolated SDA led to an increase in EPA-derived oxylipins in humans and in HepG2 cells, respectively.

**Conclusion:**

The consumption of ahiflower oil improves the circulating levels of EPA and EPA-derived oxylipins in humans. In cultivated hepatocytes, EPA and EPA-derived oxylipins are more effectively increased by SDA than ALA.

## 1 Introduction

The role of dietary n3- and n6- long chain polyunsaturated fatty acids (LC-PUFAS) in human health and disease has been studied for decades. It is generally accepted that PUFAS are involved in inflammatory processes, while n3-PUFAS are rather anti-inflammatory, n6-PUFAS exhibit rather pro-inflammatory activities (1). The World Health Organization (WHO) recommends a dietary intake of PUFAS in a n6/n3-ratio of approximately 4:1, however, the level consumed in a typical Western diet is much higher (2, 3). This disproportion may be partly responsible for the increasing incidence of chronic inflammatory diseases such as cardiovascular diseases (4, 5) or rheumatoid arthritis (6, 7). In good accordance, higher intakes of dietary n3-LC-PUFAS are associated with lower risk for cardiovascular diseases (8, 9).

**Figure 1:**
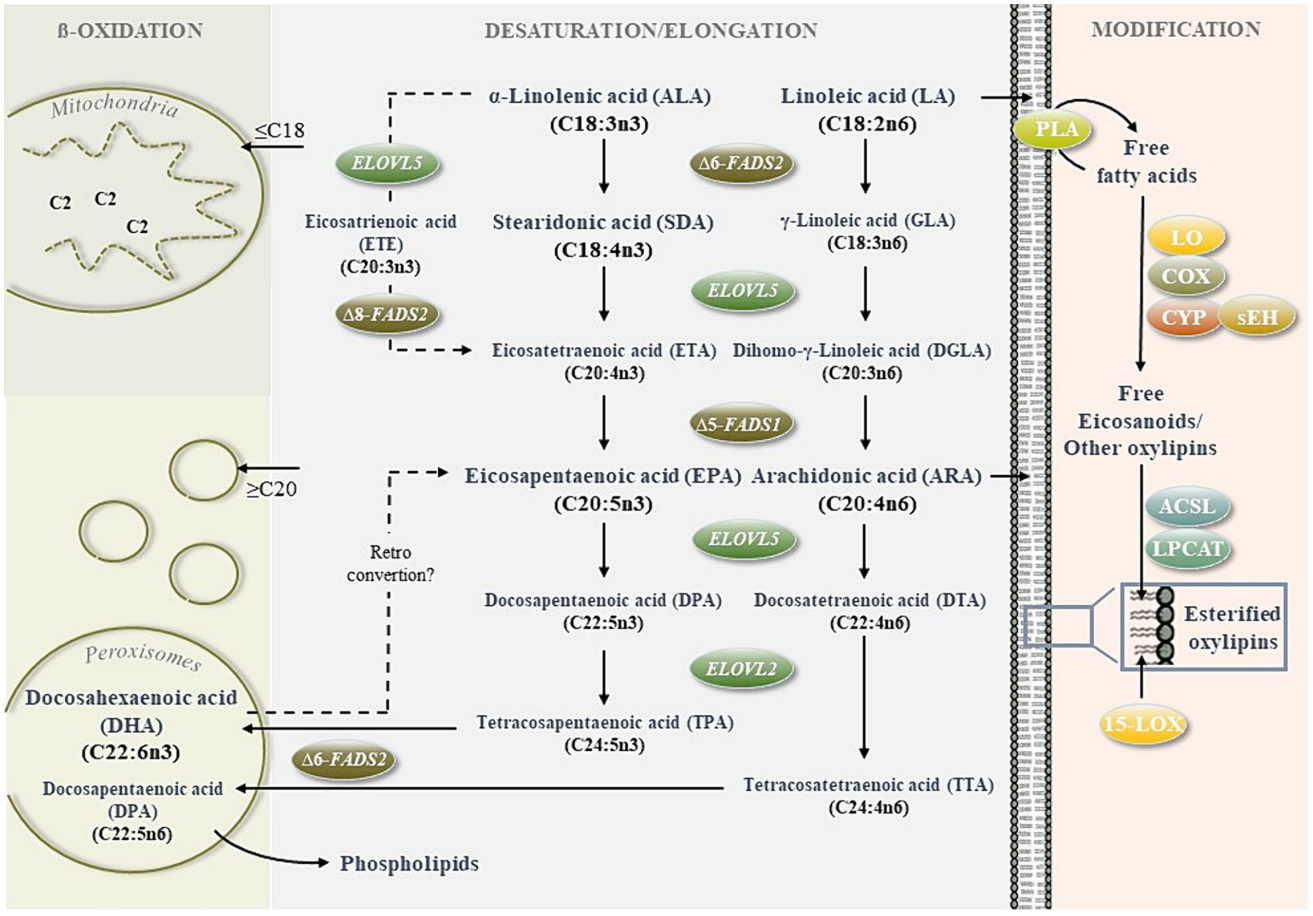
Regulation of n3- and n6 long-chain polyunsaturated fatty acid (LC-PUFA) metabolism. The cellular metabolism of LC-PUFAS is determined by synthesis (desaturation/elongation of precursor fatty acids), degradation (mitochondrial and peroxisomal ß-oxidation) and further modification to form bioactive metabolites (e.g., oxygenated lipid mediators, lipid peroxidation products).For the *de novo* synthesis of n3-PUFAS eicosapentaenoic acid (EPA) and docosahexaenoic acid (DHA) and the n6-PUFA arachidonic acid (ARA), the essential n3 and n6 precursor fatty acids α-linolenic acid (ALA) and linoleic acid (LA) must be obtained through the diet. Further conversion of ALA and LA competes for the same fatty acid desaturases and elongases. Therefore, the fatty acid desaturase FADS2 plays a key role by inserting the initial double bonds at the ∆6 and ∆8 positions (14). C2-shortening of long (≥C16) and very long (≥C20) fatty acids via ß-oxidation occurs within a peroxisomal and mitochondrial crosstalk (15). The peroxisomes preshorten fatty acids ≥ C20, which are incorporated within cells (e.g., DHA and DPA in cell membrane phospholipids) or further degraded within the mitochondria to provide energy. Oxylipins are generated mainly upon the release of PUFAS from membranes by lipases, such as phospholipase A_2_ (PLA_2_) and oxidation by lipoxygenases (LOX), cyclooxygenases (COX), cytochrome P450 monooxygenases (CYP) and soluble epoxide hydrolase (sEH). Non-esterified oxylipins can be confiscated by integration into membrane phospholipids driven by acyl-CoA synthetase long-chain family members (ACSL) and lysophosphatidylcholine acyltransferase (LPCAT)0.15-LOX can also convert esterified lipids. Moreover, it should be noted that oxylipins can be formed nonenzymatically by autoxidation.

The most important n3-LC-PUFAS are eicosapentaenoic acid (EPA, C20:5n3) and docosahexaenoic acid (DHA, C22:6n3), which mainly occur in fatty fish, such as mackerel (*Scomber scombrus*), herring (*Clupea harengus*), tuna (*Thunnus thynnus*) and salmon (*Salmo salar*) (10). However, as marine fish resources are decreasing and the global population is increasing, renewable, plant-derived n3-LC-PUFA-rich alternatives are needed to provide adequate sources of these health-relevant fatty acids. An alternative approach is to obtain EPA- and DHA from their precursors through endogenous synthesis rather than directly through food. Since the endogenous synthesis of n3-LC-PUFA (Figure 1) is strongly affected by substrate levels and the activities of rate-limiting enzymes (11), adding precursor fatty acids to food along with molecules that stimulate their enzymatic conversion could be an important strategy to improve EPA- and DHA levels in humans. Notably, potential precursor fatty acids for EPA and DHA synthesis are found in many plant-derived oils. Flaxseed oil from *Linum usitatissimum*, for example, consists of ~60% α-linolenic acid (ALA, 18:3n3). Other seed plant oils, such as echium oil (*Echium plantagineum*), lappula oil (*Lappula squarrosa*) or ahiflower oil (*Buglossoides arvensis*) are rich in both, ALA and stearidonic acid (SDA, C18:4n3) comprising 30–40% and 10–20% of the total fatty acids, respectively (12, 13). In theory, SDA bypasses the first ∆6-desaturase (FADS2)-driven desaturation step (Figure 1), which has been considered as rate-limiting within the LC-PUFA synthesis cascade (16–18). In addition, it has been shown in humans that circulating and cellular EPA levels are increased more substantially by ALA- and SDA-rich ahiflower oil than by flaxseed oil that contains only ALA (13). The stimulation of n3-LC-PUFA synthesis by plant bioactives has been studied in cultivated cells *in vitro* (14, 19), and *in vivo* in fish [Schiller (20, 21)], chickens (22) and humans (23).

Oxygenation of n3- and n6-PUFAs results in bioactive oxylipins which are known for their anti- or pro-inflammatory mode of action, respectively (24). Enzymatic routes for oxylipin synthesis are catalyzed by lipoxygenases (LOX), cyclooxygenases (COX), cytochrome P450 monooxygenases (CYP450) and soluble epoxid hydrolase (sEH) while resulting metabolites are mainly hydroxy-, epoxy- and dihydroxy-PUFAS (25, 26). Oxylipins can be esterified with phospholipids in membranes or are non-esterified (27). Well known oxylipins such as prostaglandins, thromboxanes and leucotrienes are classical eicosanoids formed from C20 fatty acids (i.e., ARA) which exhibit a wide variety of biological activities (28, 29). For several n3-PUFA oxylipins, such as EPA derived 15-HEPE, anti-inflammatory properties have been described (30). However, the formation, signaling and occurrence and thus the biological roles of the so-called specialized lipid mediators (SPM) or lipoxins are rather unclear (31).

In marine food sources, high EPA and DHA levels are usually accompanied by high amounts of taurine, an amino sulfonic acid (32). Furthermore, taurine has the potential to influence lipid adsorption and metabolism, either as a constituent of bile salts (33, 34) or as a protector of susceptible LC-PUFAs against lipid peroxidation (35–37). The present study adopts a novel approach to investigate whether taurine, either alone or in combination with n3-LC-PUFA precursors, serves as a modulator of the fatty acid pattern in humans and cultivated cells. In humans, the precursor fatty acids and taurine were orally supplied using ahiflower oil and standardized taurine capsules. In parallel, cultivated hepatocytes were treated with isolated ALA and SDA as precursors and EPA and DHA as positive controls. HepG2 cells were chosen for this purpose due to their human origin. Furthermore, HepG2 hepatocytes express desaturases and elongases necessary for the conversion of ALA and SDA to EPA and DHA (38, 39). Previous studies conducted by our group have demonstrated the feasibility of modifying the fatty acid profile through the supplementation of fatty acids and bioactives in the culture media (19). However, to the best of our knowledge, it has not yet been demonstrated experimentally whether equimolar ALA and SDA treatments of cultivated hepatocytes result in differential cellular EPA and DHA accumulation. Furthermore, there is no existing literature data regarding the impact of SDA or ahiflower oil on cellular and circulating oxylipins. The alterations in the fatty acid pattern induced by both *in vivo* and *in vitro* treatments prompted an investigation into whether these changes were similarly reflected in an altered oxylipin pattern. Overall, the present study provides novel insights into the capacity of plant derived n3-PUFAS to modulate fatty acid and oxylipin patterns in humans and cultivated cells.

## 2 Materials and methods

### 2.1 Human intervention study

#### 2.1.1 Study population

For the human intervention study, 30 healthy male volunteers aged between 20 and 31 years were recruited in September 2019. Young male volunteers in a narrow age range were selected to ensure a healthy and homogeneous study population. Females were excluded from the study because the menstrual cycle potentially affects hormone-sensitive lipid metabolism. Subjects with chronic diseases, hypertension, allergies and smokers were excluded from the study. Furthermore, consumption of fatty fish, seafood, energy drinks more than once a month and the intake of fish oil or taurine-containing supplements were considered as exclusion criteria. The initial characteristics of the study participants are shown in Table 1.

**Table 1:** Initial characteristics of the study population.

Parameter	Mean ± SD	Min - Max
Age (yr)	24.9 ±3.3	20–31
Weight (kg)	78.5 ±9.3	63.8–110.9
Body-Mass-Index (kg/m^2^)	23.8 ±2.4	19.9–30.7
Fat-Mass-Index (kg/m^2^)	4.7 ±2.1	1.1–10.7
Triacylglycerides (mmol/L)	1.21 ±0.64	0.67–3.31
Total cholesterol (mmol/L)	3.74 ±0.65	2.64–5.47
LDL cholesterol (mmol/L)	2.34 ±0.60	1.30–3.69
HDL cholesterol (mmol/L)	1.20 ±0.26	0.74–1.85
Aspartate aminotransferase (μmol/sL)	0.32 ±0.15	0.15–0.84
Alanine aminotransferase (μmol/sL)	0.39 ±0.09	0.24–0.66
Interleukin-6 (pg/mL)	< 2.00	
Tumor necrosis factor-α (pg/mL)	6.35 ±0.25	4.00–9.30
C-reactive protein (mg/L)	0.61 ±0.57	0.30–2.57
Fibrinogen (g/L)	2.37 ±0.35	1.71–3.12
n3-Index (%)^#^	3.49 ± 1.11	1.73–6.29
Systolic blood pressure (mmHg)	126.9 ±6.3	114.7–144.7
Diastolic blood pressure (mmHg)	82.6 ±4.0	76.0–92.7

^#^n3-Index of erythrocytes = docosahexaenoic acid + eicosapentaenoic acid/total fatty acids x 100.

The necessary sample size was calculated using the tool G*Power (version 3.1.9.4.). To this end, a minimal expected change in plasma EPA level of 50% and a standard deviation of 50% were assumed. The level of significance was set at 0.05 with a desired power of 0.90. The calculated minimal required sample size was 23, which was increased to 30 volunteers to compensate for potential dropouts and noncompliance. The study protocol was approved by the ethics committee of the medical faculty, of the University of Kiel Germany (D 484/18) and registered with the DRKS-ID: DRKS00018872. All subjects provided written informed consent before participation.

#### 2.1.2 Study protocol

**Figure 2:**
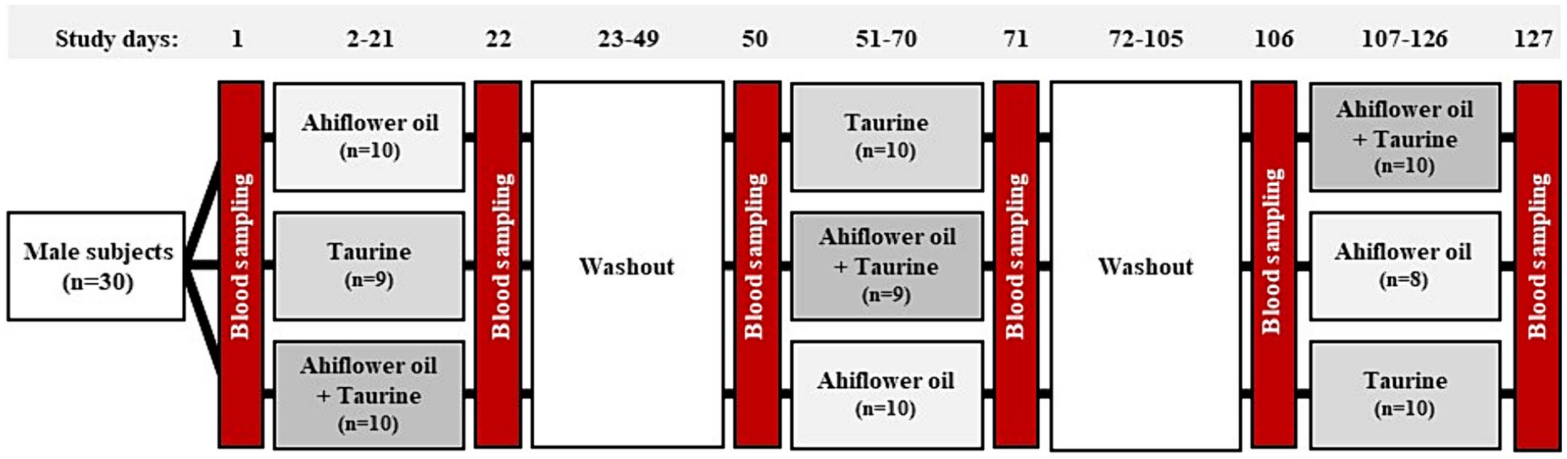
Study protocol for the crossover human intervention study. Thirty male volunteers were randomly divided into three groups, each undergoing three interventions of 20 days in a different order. Blood sampling occurred before and after each intervention phase, with washout phases of at least 26 days included between interventions. There was one dropout shortly after the study commenced, and a second dropout occurred during the final intervention phase.

The study was conducted as a monocentric, randomized crossover design at the Institute of Human Nutrition at the University of Kiel. As shown in Figure 2, participants were assigned randomly to three blocks (*n* = 10) within which treatments were administered in three different sequences lasting 20 days each. A washout phase of at least 26 days was included between interventions. The treatments were (1) ahiflower oil, (2) taurine and (3) ahiflower oil + taurine. After two dropouts at different time points, the final number of replicates (n) was 28 for ahiflower oil, 29 for taurine, and 29 for ahiflower oil + taurine. Ahiflower oil and taurine were provided in vegan capsules containing 750 mg of ahiflower oil/capsule (Nature’s Crops AHIFLOWER**®** Seed Oil, Product Code: AHI-O-NCI-1-014, Nature’s Crops International, United States) and 500 mg of taurine/capsule (Product code: 09238281, NUTRITHEKE GmbH, Röttenbach, Germany). A representative fatty acid composition of ahiflower oil is presented in Appendix 1. During the intervention phases the participants were instructed to ingest 4 capsules of ahiflower oil or/and 1 capsule of taurine with each meal (breakfast, lunch, dinner), resulting in a total daily intake of 9 g ahiflower oil and/or 1.5 g taurine. Compliance was tested at the end of each intervention period by counting the residual capsules. Participants were instructed to refrain from consuming marine foods and energy drinks during the study, but to continue normal exercise and eating habits.

#### 2.1.3 Blood sampling, body composition and blood pressure

Blood was sampled and the body composition and blood pressure were measured before (T_0_) and after (T_20_) each of the three intervention periods. The T_0_ and T_20_ days started at 07:00 am at the Institute of Human Nutrition after the participants conducted an overnight fast.

EDTA-plasma samples were used to analyze fatty acids and oxylipins and serum samples to analyze of triacylglycerides (TAG), total cholesterol (CHOL), low density lipoproteins (LDL), high density lipoproteins (HDL), aspartate aminotransferase (ASAT), alanine aminotransferase (ALAT), interleukin 6 (IL-6), tumor necrosis factor α (TNF-α) and C-reactive protein (CRP). Citrat-Plasma was used for the analysis of fibrinogen (FIB).

Body composition was analyzed by air displacement plethysmography using the BOD POD device (Cosmed, Rome, Italy) to determine fat mass (FM) and fat free mass (FFM). Furthermore, an electronic scale coupled to the BOD POD device was used for weight assessment. Blood pressure was measured with a sphygmomanometer (boso, BOSCH + SOHN GmbH & Co KG, Jungingen, Germany).

### 2.2 Cell culture studies

HepG2 cells (Institut für Angewandte Zellkultur, Munich, Germany) were cultured in RPMI medium (RPMI-1640, P04-18047, PAN™ Biotech GmbH) supplemented with 10% fetal bovine serum (FBS, Thermo Fisher Scientific) and 1% penicillin/streptomycin (PAN™ Biotech GmbH). Cells were grown at 37°C in a humidified 5% CO_2_ incubator. For experiments, cells (at passage numbers 5–13) were seeded in respective cell culture plates and following a 24 h growth phase, medium was switched to FBS-free starvation medium. After 24 h of starvation, HepG2 cells were treated with taurine and isolated fatty acids for additional 24 h before being harvested by scraping in 200 μL of 0.2 mmol/L sodium chloride. Specifically, the treatments included 1 mM taurine, 50 μmol/L of isolated fatty acids (ALA, SDA, EPA, or DHA), and a combination of 1 mM taurine and 50 μmol/L SDA. As taurine is water-soluble, RPMI medium served as the control. Stock solutions of fatty acids were prepared in EtOH, covered with nitrogen, and stored at −80°C until use. A final EtOH concentration of 0.1% was used as the vehicle control for fatty acids. The n3-PUFAS ALA, EPA and DHA as well as taurine were purchased from Sigma Aldrich (Merck KGaA, Darmstadt, Germany). When the experiments were conducted, SDA was not commercially available. Therefore, SDA was isolated from the seed oil of *Lappula squarrosa*, as described below.

### 2.3 Isolation of stearidonic acid via countercurrent chromatography

SDA was isolated from *Lappula squarrosa* (Exsemine GmbH Salzatal, Germany) plant oil containing 19.4% SDA. The oil was saponified with ethanol and aqueous potassium hydroxide for total fatty acid extraction. Afterwards, free fatty acids were separated by high performance countercurrent chromatography (HPCCC) according to Englert and Vetter (40). The parameters for HPCCC separation of fatty acids are shown in Table 2.

**Table 2:** Conditions used for fatty acid separation via high performance countercurrent chromatography.

Modus	Head-to-Tail; co-current
Coil volume	125,5 mL
Solvent system	Hexane/ACN 1/1 *v/v*
Stationary phase	Upper phase
Mobile phase	Lower phase
Sample	Free fatty acids of *Lappula squarrosa* (Exsemine GmbH Salzatal)
Injection amount	170 mg
Rotation	860 rpm
Flow rate	3 mL/min 2 mL/min lower phase 1 mL/min upper phase
Injection volume	5 mL
Retention	91%

The fractions were methylated using methanolic sulfuric acid and FAMES were analyzed by gas chromatography (GC) as shown in Table 3. Three out of the 14 fractions contained SDA with chromatographic purities of 47, 98, and > 99%. Cell culture studies were conducted using the fraction containing >99% SDA.

**Table 3:** Gas chromatography analysis of fatty acid methyl esters.

Device	TRACE 1300 Gas Chromatograph, Thermo Fisher Scientific (Waltham, Massachusetts, USA)
Autosampler	Al 1,310, Thermo Fisher Scientific (Waltham, Massachusetts, USA)
Injector	Front SSL, Temperature: 240°C, Split 1:5
Column	DB-FastFAME; Internal diameter (0.25 mm); length (30 m); Solid phase thickness (0.25 μm); Agilent Technologies (Santa Clara, California, USA)
Carrier gas	Helium (1,200 mL/min)
Detector	FID, temperature: 240°C
Detector gas	Hydrogen (35 mL/min), synthetic air (350 mL/min)
Make-up gas	Nitrogen (40 mL/min)
Data processing	Chromeleon®; version 7.2.9
Temperature program	50°C (1 min) 50°C - 175°C, 65°C/min 175°C - 185°C, 10°C/min (0,5 min) 185°C - 220°C, 2°C/min (2,0 min)
Internal standard	C 17:0; C 19:0; C 21:0

### 2.4 Analytics

#### 2.4.1 Analysis of clinical biomarkers

Clinical biomarkers, such as TAGS, CHOL, LDL, HDL, ASAT, ALAT, IL-6, TNF-α, CRP, and FIB, underwent analysis at a service medical laboratory accredited in accordance with DIN EN ISO 15189 standards (Labor MVZ Westmecklenburg, Schwerin, Germany).

#### 2.4.2 Fatty acid analysis

Fatty acid composition was determined in 100 μL ETDA-plasma derived from humans and 100 μL HepG2 suspension. The extraction and derivatization procedure was conducted according to a detailed protocol described previously (41). Briefly, samples were stabilized with an antioxidant solution containing ethylenediamintetraacetic acid (EDTA) and butylated hydroxytoluene (BHT), hydrolysed with 10 mmol/L aqueous sodium hydroxide and neutralized with 50% acetic acid. Afterwards, fatty acids were extracted with tert-butyl methyl ether/methanol (MTBE/MeOH). Solvents were evaporated and the lipid pellet was redissolved in acetylchloride/methanol and n-hexane. The mixture was heated in crimp vials for 1 h at ~90°C to form FAMES. After cooling, the solution was transferred to 0.44 mol/L potassium carbonate and the upper hexane phase was used for FAMES analyses via gas chromatography (conditions are summarized in Table 3). Fatty acid composition was calculated as a percentage of the total identified FAMEs.

Relative values of selected fatty acids were calculated as % of total FAMES in plasma before (T_0_) and after (T_20_) 20 days of (1) ahiflower oil, (2) taurine and (3) ahiflower oil + taurine intervention. Intervention effects were calculated as the difference between T_0_ and T_20_ least squares means (△LSmeans). As described below, the comparisons between △LSmeans were used to interrogate treatment significance.

#### 2.4.3 Quantification of non-esterified oxylipin concentrations in plasma and cell samples

The non-esterified oxylipin concentrations in the plasma of subjects following ahiflower oil intervention and cell culture experiments were determined as described elsewhere (42–44). For the analysis, 500 μL plasma or 400 μL of homogenized cells were mixed with antioxidant and inhibitor solution and deuterated internal standards. Following protein precipitation with methanol at −80°C and centrifugation, the supernatant was purified by solid-phase extraction (43). The cell pellet was redissolved in 2% (*w/v*) sodium dodecyl sulfate and used for protein content determination via bicinchoninic acid assay (42). The extracts were analyzed using a liquid chromatography 1,290 Infinity II LC system (Agilent, Waldbronn, Germany) coupled to a triple quadrupole mass spectrometer QTRAP 5500 (AB Sciex, Darmstadt, Germany) as previously described (42–44). Quantification was carried out by external calibration with internal standard.

### 2.5 Statistical analysis

Data from the human intervention study were analyzed as a mixed-effect model using SAS statistical package (SAS Institute). Before the analysis, the differences between T_0_ and T_20_ were calculated for all variables and used as metrics to interrogate treatment effects after the normality was examined using the univariate procedure of SAS. With the exemption of oxylipins, the model included the fixed effect of the intervention, treatment sequence, and period, as well as the random effect of the participant nested within treatment sequence. Delta least squares means (**△**LSmeans) were compared among treatments using Fisher’s least significant difference test. If the sequence effect was significant for a variable, then carryover effects presumably occurred and, consequently, only the first period was used for analysis. In this case, the model was simplified by removing the effects caused by treatment sequence and period from the model. The same approach was used for analyzing the impact of ahiflower oil on circulating oxylipins, although the model included fixed effects, i.e., the sampling time (T_0_ and T_20_) and period, as well as the random effect of the participant.

Statistical analyses for cell culture data were conducted using GraphPad PRISM 9 software (San Diego, CA, United States). If data passed the normality of the distribution test (Kolmogorov–Smirnov and Shapiro–Wilk tests, *p* > 0.05), a one-way analysis of variance (ANOVA incl. Tukey *post hoc* test or Welch ANOVA incl. Dunnett’s T3 *post hoc* test) for multiple comparisons was then carried out. If only two variables were compared a parametric t-test with Welch’s correction for two treatment comparisons was used. If the data did not pass the normality test (*p*-value <0.05), the Kruskal Wallis test (multiple comparisons) or nonparametric t-test (two comparisons) was used for significance analysis.

## 3 Results

### 3.1 Human intervention study

As summarized in Table 4, supplementation with ahiflower oil or with ahiflower oil + taurine markedly affected the fatty acid composition of human plasma. However, taurine treatment did not alter fatty acid levels when ingested alone and did not modify the ahiflower effects when ingested with the oil. Consumption of ahiflower oil at least doubled plasma concentrations of ALA (1.1 to 2.3) and SDA (0.2 to 0.5), which was expressed as the % of total FAMEs. Furthermore, plasma levels of the target fatty acid EPA were tripled (0.2 to 0.6) following the ahiflower oil ingestion, while DHA levels (3.3 to 2.5) were not significantly changed in response to the ahiflower oil interventions. The total of n3 PUFAS (∑n3 PUFA) was significantly higher only after supplementation with ahiflower oil + taurine and showed a tendency to increase (*p* = 0.08) following consumption of ahiflower oil alone. The total of n6 PUFAS (∑n6 PUFA) was not affected by the treatments. However, individual n6/n3 indices were highly heterogenous with means for ahiflower oil (T0 = 27.8 and T20 = 14.3), taurine (T0 = 28.7 and T20 = 24.9) and ahiflower oil + taurine (T0 = 27.7 and T20 = 13.0). The complete dataset for individual ∑n6 PUFAs, ∑n3 PUFAs and n6/n3 indices is presented in Appendix 2.

**Table 4:** Fatty acid composition (% of total fatty acid methyl esters) in human plasma before (T_0_) and after (T_20_) 20 days of ahifloweroil, taurine and ahifloweroil + taurine intervention.

	Ahifloweroil (*n* = 28)			Taurine (*n* = 29)			Ahifloweroil + Taurine (*n* = 29)		
	T_0_	T_20_	T_20_-T_0_	T_0_	T_20_	T_20_-T_0_	T_0_	T_20_	T_20_-T_0_
16:0	7.7 (4.8–10.6)	5.9 (3.4–8.3)	−2.0	7.3 (4.7–9.9)	7.8 (4.7–10.9)	0.6	7.9 (5.2–10.6)	6.2 (3.7–8.8)	−1.7
18:0	3.3 (2.2–4.4)	2.5 (1.5–3.6)	−0.8	3.5 (2.4–4.7)	3.0 (1.9–4.2)	−0.5	3.3 (2.2–4.4)	2.8 (1.8–3.8)	−0.5
Σ SFA	13.0 (8.5–17.4)	11.0 (6.5–15.4)	−2.3	12.5 (8.5–16.6)	13.3 (8.2–18.5)	1.0	13.0 (8.7–17.2)	11.8 (7.3–16.1)	−1.2
16:1n7	0.6 (0.3–0.8)	0.4 (0.2–0.6)	−0.2	0.7 (0.4–0.9)	0.6 (0.3–0.9)	−0.09	0.6 (0.3–0.8)	0.5 (0.3–0.7)	−0.08
18:1n9	25.7 (22.7–28.7)	23.2 (20.9–25.6)	−2.6	24.6 (21.4–27.9)	24.6 (21.3–27.9)	−0.3	24.8 (21.9–27.7)	23.6 (21.0–26.2)	−1.2
Σ MUFA ^PE^	27.3 (24.6–29.9)	24.7 (22.8–26.5)	−2.7	26.2 (22.7–29.8)	26.0 (23.1–28.9)	−0.4	26.3 (23.7–28.8)	25.0 (22.8–27.2)	−1.3
18:2n6	50.1 (43.2–57.1)	54.6 (49.0–60.1)	4.9	52.7 (47.5–57.9)	51.1 (45.0–57.1)	−1.7	52.4 (47.7–57.1)	52.9 (47.7–58.1)	0.5
18:3n3	1.1 (0.9–1.3)	2.3 (2.0–2.5)	1.1^*a^	1.0 (0.9–1.2)	1.1 (0.9–1.3)	0.07^b^	1.1 (0.9–1.2)	2.2 (1.8–2.4)	1.1^* a^
18:4n3	0.2 (0.1–0.3)	0.5 (0.4–0.7)	0.3^*a^	0.2 (0.08–0.2)	0.2 (0.1–0.2)	0.02^b^	0.2 (0.1–0.2)	0.6 (0.5–0.7)	0.4^*a^
20:3n6	0.6 (0.4–0.8)	0.6 (0.3–0.8)	−0.05	0.5 (0.3–0.7)	0.6 (0.3–0.8)	0.08	0.6 (0.3–0.8)	0.5 (0.3–0.7)	−0.03
20:4n6	3.1 (2.0–4.2)	2.3 (1.3–3.4)	−0.9	2.7 (1.6–3.7)	3.0 (1.9–4.2)	0.4	2.7 (1.7–3.7)	2.7 (1.7–3.7)	0.05
20:5n3	0.2 (0.1–0.3)	0.6 (0.4–0.9)	0.4^*a^	0.3 (0.1–0.4)	0.3 (0.2–0.4)	0.04 ^b^	0.3 (0.1–0.4)	0.7 (0.4–1.0)	0.4^*a^
22:6n3	3.3 (2.0–4.6)	2.5 (1.4–3.6)	−0.8	3.2 (1.7–4.7)	3.7 (1.5–5.9)	0.6	2.9 (1.7–4.1)	2.9 (1.8–4.0)	0.02
Σ n6 PUFA	54.3 (48.4–60.2)	57.9 (53.6–62.2)	3.6	56.2 (51.7–60.6)	54.9 (50.3–59.6)	–1.2	55.9 (52.3–59.6)	56.5 (52.4–60.6)	0.6
Σ n3 PUFA	4.8 (3.4–6.2)	6.0 (4.7–7.2)	1.2	4.7 (3.1–6.1)	5.3 (3.1–7.5)	0.7	4.4 (3.2–5.7)	6.4 (5.2–7.6)	2.0^*^

The sum of saturated fatty acids (ΣSFA), mono unsaturated fatty acids (ΣMUFA) and polyunsaturated fatty acids (ΣPUFA) were calculated from the whole dataset of fatty acids, which is not completely listed in the table. Data are shown as the means with lower and upper limits of the 95% confidence interval in brackets. Intervention effects (T_20_- T_0_) are expressed as △ of least square means (△ LSmeans). Asterisks indicate significant intervention effects between T0 and T20. Different letters indicate significant differences between interventions. PE indicates a period effect, and only the first period was used for statistical analysis.

**Figure 3:**
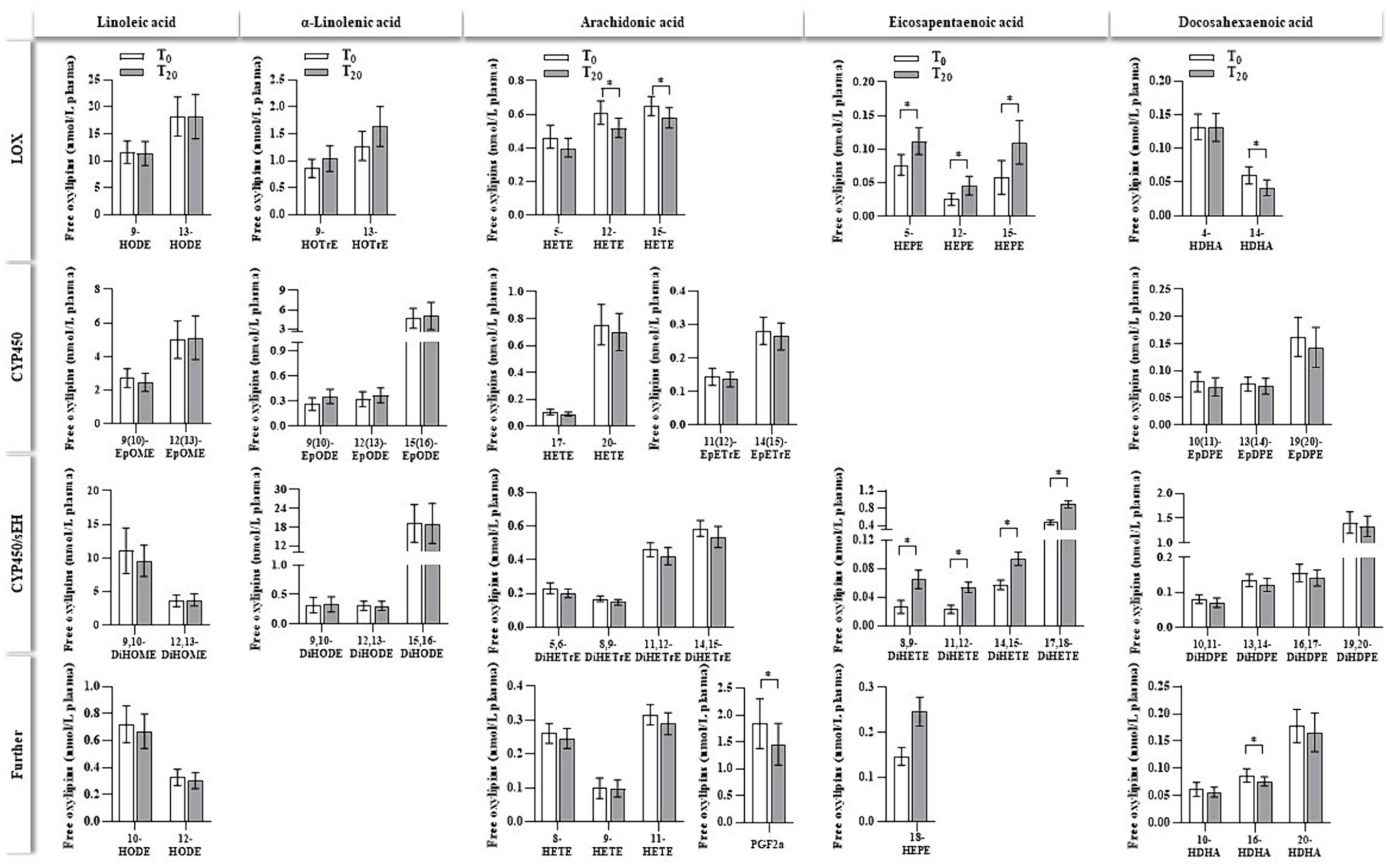
Non-esterified oxylipins (nmol/L plasma) in human plasma before (T0) and after (T20) 20 days of ahiflower oil intervention. Oxylipins are grouped based on their precursor fatty acid and enzymatic formation routes, which are catalyzed by lipoxygenase (LOX), cytochrome P450 enzyme (CYP450), CYP450/soluble epoxide hydrolase (CYP450/sEH) or other enzymes (further). Notably, several oxylipins can be formed by different enzymes as well as by autoxidation (e.g., 15-HETE can be formed by LOX, COX, CYP and autoxidation). Data are shown as the means with the lower and upper 95% confidence intervals (*n* = 28). * indicate significant intervention effects (*p* < 0.05), which were determined by comparing LSmeans of T_0_ vs. T_20_ using a mixed-effect model.

For the additional measurement of non-esterified oxylipin levels, only plasma obtained from the ahiflower oil intervention was utilized. As depicted in Figure 3, non-esterified oxylipins were detected in plasma before (T_0_) and after (T_20_) the ahiflower oil intervention. The results were grouped via enzymatic synthesis routes by (1) lipoxygenases (LOX), (2) cytochrome P450 oxidase enzymes (CYP450), (3) CYP450/soluble epoxid hydrolase (sEH) and (4) further enzyme reactions or autoxidation. Detected oxylipins derived from LA, ALA, ARA, EPA and DHA were analyzed, while measurements of SDA-derived oxylipins are not included in the instrumental method because synthetic standards are lacking (42). Ahiflower oil ingestion led to a slight but significant decrease in several ARA-derived eicosanoids, such as 12-HETE, 15-HETE and PGF2a. EPA- derived eicosanoids were increased in response to ahiflower oil intake. For DHA, the ahiflower oil intervention tended to marginally decrease the circulating levels of its oxygenation products, which was significant for 14-HDHA and 16-HDHA.

**Figure 4:**
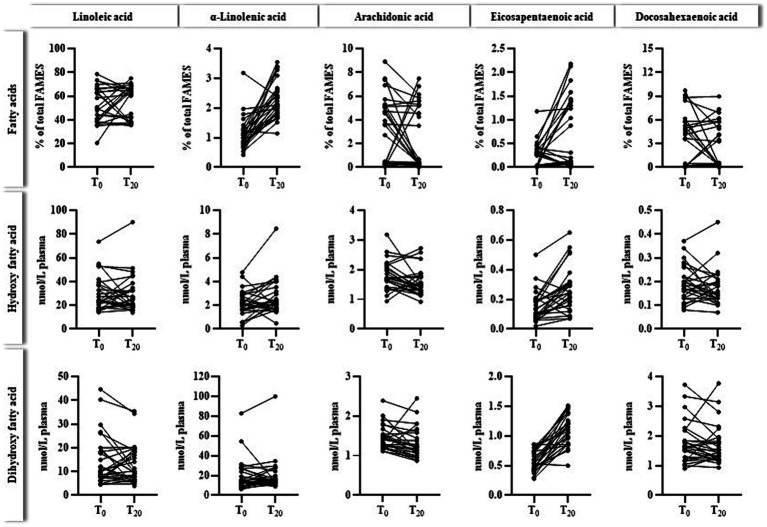
Individual levels (*n* = 28) of plasma fatty acids (% of total FAMEs) and the sum of corresponding hydroxy- and dihydroxy fatty acids (nmol/L plasma) before (T_0_) and after (T_20_) the ahiflower oil intervention. The cumulated levels of oxylipins were calculated as follows: hydroxy fatty acids for linoleic acid (9-HODE, 13-HODE), α-linolenic acid (9-HOTrE, 13-HOTrE), arachidonic acid (5-HETE, 12-HETE, 15-HETE), eicosapentaenoic acid (5-HEPE, 12-HEPE, 15-HEPE) and docosahexaenoic acid (4-HDHA, 14-HDHA), dihydroxy fatty acids for linoleic acid (9,10-DiHOME, 12,13-DiHOME), α-linolenic acid (9,10-DiHODE, 12,13-DiHODE, 15,16-DiHODE), arachidonic acid (5,6-DiHETrE, 8,9-DiHETrE, 11,12-DiHETrE, 14,15-DiHETrE), eicosapentaenoic acid (9,10-DiHETE, 11,12-DiHETE, 14,15-DiHETE, 17,18-DiHETE) and docosahexaenoic acid (10,11-DiHDPE, 13,14-DiHDPE, 16,17-DiHPDE, 19,20-DiHPDE).

Figure 4 outlines the relation between plasma levels of selected n3- and n6- PUFAS and their corresponding oxylipins before (T_0_) and after (T_20_) ahiflower oil intervention. As also shown in Table 4 plasma levels of ALA and EPA usually increased due to the ahiflower oil intake. Regarding the primary target EPA, a few participants did not respond to the intervention. For oxylipins, only EPA-derived oxylipins increased after 20 days of ahiflower oil ingestion, which was clearly indicative of dihydroxy-EPA metabolites.

### 3.2 Studies with cultivated hepatocytes

Taurine treatment did not affect the fatty acid composition of HepG2 cells compared to RPMI control cells (Table 5). In contrast, incubation with n3 fatty acids significantly increased the cellular concentration of the respective fatty acid, while the final accumulation in % of total FAMES increased from SDA (1.7) < ALA (5.7) < EPA (9.2) < DHA (16.6). ALA and SDA incubation generally increased total cellular PUFAs compared to EtOH treated cells. The cellular contents of n6-PUFAS LA and ARA were not affected, while n3-PUFAS increased considerably. Consequently, the cellular n6/n3-index was much higher for cells treated with EtOH (2.2) than for ALA (0.3) and SDA (0.4) treated cells. Only ALA-incubated cells accumulated detectable levels of eicosatrienoic acid (ETA). SDA led to significantly higher EPA levels compared to ALA supplementation (5.1 vs. 3.0% of total FAMEs), while DHA levels were unaffected by ALA and SDA. Coincubation of SDA and taurine did not alter the effects observed by SDA.

**Table 5:** Fatty acid composition (% of total fatty acid methyl esters) of HepG2 cells after 24 h of treatment with different n3 fatty acids and taurine.

	RPMI	Taurine	EtOH	ALA	SDA	SDA + Taurine	EPA	DHA
16:0	31.4 (29.9–32.9)	32.7 (32.0–33.4)	31.6 (30.5–32.7)^ab^	31.6 (30.5–32.6)^ab^	32.4 (31.7–33.1)^a^	32.8 (29.9–35.7)^a^	32.5 (30.7–34.4)^a^	29.7 (28.3–31.0)^b^
18:0	10.1 (9.7–10.6)	11.3 (9.3–13.2)	10.5 (10.1–11.0)	11.4 (10.8–12.1)	11.5 (10.5–12.4)	11.0 (10.5–11.5)	11.8 (11.1–12.5)	10.6 (10.2–11.1)
Σ SFA	44.5 (42.8–46.2)	46.8 (44.2–49.4)	44.9 (43.7–46.0)^ab^	47.1 (43.3–50.9)^ab^	47.4 (46.3–48.5)^ab^	46.9 (43.7–50.0)^ab^	48.5 (44.8–52.2)^a^	43.8 (41.7–45.9)^b^
16:1n7	8.1 (7.9–8.4)	8.2 (7.3–9.1)	8.4 (8.1–8.7)^a^	6.8 (6.5–7.1)^b^	7.4 (7.0–7.8)^c^	7.8 (7.2–8.5)^c^	6.4 (5.9–6.9)^be^	6.9 (6.8–7.0)^bce^
18:1n9	35.5 (34.1–36.9)	33.9 (33.0–34.8)	35.4 (34.1–36.7)^a^	27.3 (25.0–29.7)^b^	28.7 (27.2–30.2)^b^	30.3 (25.5–35.1)^ab^	26.1 (22.9–29.3)^b^	26.4 (24.1–28.8)^b^
Σ MUFA	46.0 (43.9–48.0)	44.0 (42.0–46.0)	45.9 (44.6–47.3)^a^	35.6 (32.9–38.2)^b^	37.7 (36.0–39.3)^b^	39.9 (35.3–44.5)^ab^	34.2 (30.1–38.2)^b^	34.9 (32.0–37.8)^b^
18:2n6	0.9 (0.7–1.1)	0.9 (0.8–1.0)	0.9 (0.7–1.0)	0.8 (0.7–0.9)	0.8 (0.7–0.9)	0.6 (0.0–1.3)	0.8 (0.7–0.9)	0.8 (0.6–0.9)
18:3n3	–	–	–	5.7 (5.2–6.1)	–	–	–	–
18:4n3	–	–	–	–	1.7 (1.6–1.9)	1.3 (0.4–2.2)	–	–
20:3n3	–	–	–	1.4 (1.3–1.4)	–	–	–	–
20:4n6	2.8 (2.4–3.2)	2.6 (2.3–2.9)	2.7 (2.3–3.0)^a^	2.3 (2.0–2.6)^b^	2.4 (2.1–2.7)^ab^	2.4 (2.2–2.6)^ab^	2.3 (2.1–2.6)^ab^	2.1 (1.8–2.4)^b^
20:5n3	-	-	-	3.0 (2.8–3.2)^a^	5.1 (4.7–5.5)^b^	4.0 (1.7–6.3)^ab^	9.2 (7.5–10.9)^c^	0.3 (0.0–0.9)^d^
22:6n3	2.2 (1.9–2.6)	2.3 (2.2–2.4)	2.2 (1.8–2.5)^a^	2.4 (2.1–2.6)^a^	2.7 (2.3–3.2)^ab^	2.6 (2.0–3.1)^ab^	3.4 (3.0–3.7)^b^	16.6 (14.2–19.1)^c^
Σ PUFA	9.5 (8.9–10.2)	9.2 (8.6–9.8)	9.2 (8.8–9.7)^a^	17.4 (16.2–18.5)^b^	14.9 (13.9–15.9)^c^	13.2 (10.3–16.2)^c^	17.4 (15.9–18.8)^b^	21.3 (19.7–23.0)^d^
n6/n3 Index	2.1 (2.0–2.2)	2.0 (1.7–2.2)	2.2 (2.0–2.3)^a^	0.3 (0.26–0.31)^bc^	0.4 (0.38–0.43)^bc^	0.5 (0.2–0.9)^b^	0.3 (0.2–0.3)^bc^	0.2 (0.15–0.22)^c^

HepG2 cells were treated with the n3- fatty acids α-linolenic acid (ALA), stearidonic acid (SDA), eicosapentaenoic acid (EPA) and docosahexaenoic acid (DHA). In parallel, taurine was added to the cells alone or in combination with SDA (SDA + Taurine). RPMI was used as a medium control for taurine-treated cells, and ethanol (EtOH) was used as vehicle control for fatty acid-treated cells. Data are shown as the means with lower and upper limits of the 95% confidence interval (n = 4). RPMI and taurine treatments were statistically compared using unpaired t-test while statistics for EtOH, ALA, SDA, SDA + Taurine, EPA and DHA were conducted via multiple comparison tests (parametric ANOVA or nonparametric Kruskal-Wallis test). Different letters indicate significant treatment effects.

**Figure 5:**
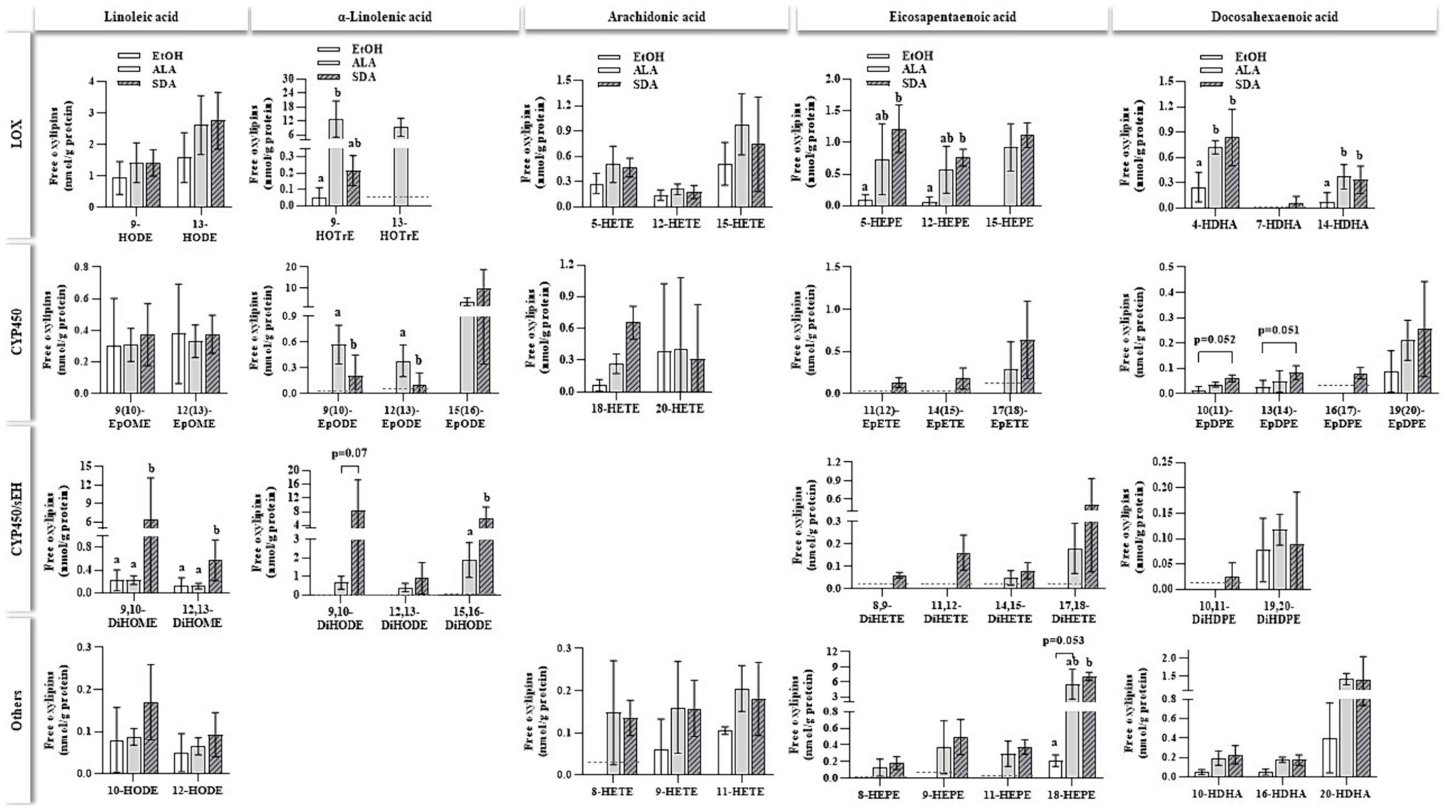
Non-esterified oxylipins levels in HepG2 cells after 24 h of incubation with different n3-fatty acids. HepG2 cells were incubated with 50 μmol/L of α-linolenic acid (ALA) and stearidonic acid (SDA). Ethanol (EtOH) was used as a vehicle control. Oxylipins (nmol/g protein) were grouped based on their precursor fatty acid and their respective formation route catalyzed by lipoxygenase (LOX), cytochrome P450 enzyme (CYP450), CYP450/soluble epoxide hydrolase (CYP450/sEH) or other pathways. Dashed lines indicate the lower limit of quantification (LLOQ) for the respective oxylipin. If <50% of values were below the LLOQ, the group was set to < LLOQ. Data are shown as the means with lower and upper limits of the 95% confidence interval in brackets (n = 4). Statistics were conducted using unpaired t-test for two groups or multiple comparison tests (parametric ANOVA or nonparametric Kruskal-Wallis test) for comparing three groups. Different letters indicate significant differences between treatments (*p* < 0.05). The complete dataset is available in Appendix 3.

Supplementation with n3-PUFAS ALA, EPA and DHA markedly increased the cellular content of their respective oxylipins compared to the EtOH vehicle control (Figure 5; Supplementary material) as well as with precursor n3- fatty acids ALA. Oxylipins were grouped regarding their precursor fatty acids as well as their synthesis route. The measurement of SDA-derived oxylipins has not been established yet. SDA treatment increased several ALA-, EPA-, and DHA-derived oxylipins. In particular, CYP and CYP/sEH-generated dihydroxy oxylipins derived from ALA were found in considerably higher concentrations in ALA- and SDA-treated cells compared to the solvent controls (<LLOQ). In contrast, ARA-derived oxylipins were not affected by ALA and SDA. A significant increase in the formation of EPA-derived oxylipins was observed in SDA (14 EPA-derived oxylipins) and ALA (7 EPA-derived oxylipins) treated cells compared to solvent controls.

## 4 Discussion

The present human intervention study clearly reveals the ability of dietary ALA and SDA-rich ahiflower oil to improve EPA status *in vivo*. Circulating EPA levels increased nearly 3-fold, while DHA levels remained largely unaffected due to the ahiflower oil intervention. It is known that in humans, plasma EPA levels are usually lower than DHA levels and respond better to dietary interventions than DHA, although there are large interindividual differences (45). The daily consumption dose of 9 g ahiflower oil accounts for an ALA and SDA intake of ~4 g/d and of ~1.7 g/d, respectively. These intake rates are thus considerably higher than those in human intervention studies involving EPA and DHA-rich oils. For example, 12 weeks of n3-PUFA supplementation with 1.4 g EPA and 0.2 g DHA per day in youth (adolescents) with mood disorders elevated EPA plasma levels nearly 4-fold, while DHA levels were only slightly affected (46). In a bioavailability study involving healthy adults, 4 weeks of daily supplementation with 816 mg of EPA and 522 mg of DHA resulted in approximately a 5-fold increase in EPA plasma levels and a 1.5-fold increase in DHA levels. This effect was observed irrespective of whether EPA and DHA were provided in the form of fish oil ethyl ester, fish oil triglycerides, or krill oil (47). In our study, the conversion rate of ALA and/or SDA to EPA was relatively high, which corresponds with other human studies involving plant-derived ahiflower (13, 48) or echium oil (49). As suggested by these results, compared to ALA-rich linseed oil, ALA and SDA-rich ahiflower (13) and echium oil (49) more greatly increase circulating and cellular EPA levels. With regard to the unaffected DHA levels, a recent study in mice, utilizing C13-labeled fatty acids derived from fish DHA, ahiflower oil and flaxseed oil, also demonstrated that even when DHA levels in serum, liver, adipose tissue, and the brain are not affected by all of the interventions, the synthesis/turnover of DHA increases over time. Moreover, diets rich in DHA and ahiflower oil exhibit similar DHA turnover rates in the liver, adipose, and brain tissues, while turnover rates from flaxseed oil were significantly slower compared to the DHA diet (50). Thus, the study suggests that measuring DHA levels may not provide as accurate an assessment of the potency of precursors for DHA synthesis as determining the synthesis/turnover rate from these precursors.

To examine the capacity of individual n3-PUFAS as precursors for EPA synthesis, we conducted additional mechanistic studies with cultured HepG2 hepatocytes. A 24 h treatment with different n3 fatty acids supplied at equal concentrations significantly increased EPA cellular concentrations, while the extent of final accumulation in % of total FAMEs increased as follows: SDA (1.7) < ALA (5.7) < EPA (9.2) < DHA (16.6). Furthermore, supplementation with ALA and SDA increased the cellular EPA concentration from zero to 3.0 and 5.1% of the total FAMEs, respectively. Under the conditions investigated, the results confirm that SDA was 65% more efficient in enhancing cellular EPA levels than ALA.

When taurine was tested as a putative modulator for the conversion of ALA/SDA to EPA/DHA *in vivo*, we found that the circulating fatty acid pattern was not affected regardless if taurine was consumed alone or in combination with ahiflower oil. Likewise, taurine treatment did not alter the n3-PUFA levels of control, ALA or SDA-supplemented HepG2 cells. Hence, our data suggest that although taurine is present in n3-PUFA-rich tissues at high concentrations such as the retina (51), it does not impact PUFA accumulation or synthesis. However, the association of PUFAS and taurine may protect PUFAS against lipid peroxidation (35–37) and support osmoregulatory functions (52). In mice, dietary EPA and DHA provision (wild-type C57BL/6 mice) increased the levels of EPA and DHA more efficiently than improved endogenous *de novo* synthesis in transgenic *fat-1* mice (53). Moreover, by employing this mouse model, a recent study showed better protection from high-fat diet induced metabolic and hepatic disorders was obtained with endogenously generated n3-PUFAs than dietary n3-PUFAs (54). Therefore, the level and origin of EPA and DHA (endogenous vs. exogenous) should be investigated.

The main synthesis routes for n3 EPA and n6 ARA utilize the same desaturases and elongases (Figure 1). Apart from its high content of n3-PUFAs, ahiflower oil also contains significant amounts of n6 linoleic acid (LA) (9–15%) which is the precursor for ARA synthesis. Interestingly, LA intake via ahiflower oil did not lead to an increase of ARA levels in human plasma but a slight yet nonsignificant decrease. In theory, the conversion of ALA and LA to form EPA and ARA compete for the same desaturases and elongases. Enhanced EPA synthesis might come at the expense of ARA synthesis. However, this observation has not been reported by others who supplemented humans with plant derived oil rich in ALA (55) and/or SDA (13, 49).

In cultivated HepG2 cells, only ALA and DHA treatment significantly decreased the cellular ARA content. However, cellular fatty acid levels were expressed in % of total FAMEs, which shift with additional accumulation of supplemented ALA and DHA. Therefore, the effects on ARA observed in humans and HepG2 cells may be negligible. The cell culture results also reveal an alternative pathway from ALA to EPA, as ALA-treated cells accumulate its elongation product ETE, but neither SDA nor ETA were found in these samples. Hence, in this cell model ALA is initially elongated to ETE by the action of ELOVL5 and is not desaturated to SDA by the Δ6-FADS2-catalyzed reaction. The conversion of radio-labeled ALA to ETE has already been achieved in cultured hepatoma 7288 C cells (56–58). Furthermore, *in vivo* studies with salmon (59) and zebrafish (60) revealed that partial knockout of the *fads2* gene resulted in the accumulation of LA and ALA C20-elongation products, which is indicative of alternative elongation prior to Δ8-FADS2-driven desaturation. Another explanation for the absence of SDA accumulation in ALA-treated cells was given by Park et al. (61), who described that SDA undergoes a coupled elongation immediately after Δ6-FADS2-driven desaturation, concomitantly restraining cellular SDA accumulation (61). In accordance with results from the current human intervention study, neither ALA nor SDA significantly increased cellular DHA levels in cultured HepG2 cells. In line with previous studies on fatty acid pattern in cultured hepatoma cell lines (19, 62), DHA was found to be the most abundant n3-PUFA in both RPMI and the vehicle control (EtOH) cells. According to Else et al. (63), FBS contains considerable amounts of DHA (~ 2–3% of total fatty acids) and in many cases also its precursor fatty acids ALA, SDA, EPA and DPA. Therefore, we hypothesize that the cellular accumulation of DHA supplied by FBS in the media and baseline synthesis of DHA from its precursors is already substantial, making further modulation challenging.

Both n3- and n6-PUFAS are precursors of bioactive eicosanoids and other oxylipins (1). Accordingly, we found that in humans, oral intake of ahiflower oil, which predominantly led to elevated plasma levels of EPA, significantly increased the concentration of EPA-derived eicosanoids in plasma, in contrast, DHA-derived oxylipins were less affected (only 14-HDHA and 16-HDHA levels were slightly decreased). This supports the assumption that the formation of oxylipins is partly determined by the content of their respective precursor fatty acids. Similar outcomes have been observed in previous feeding studies, in which dietary intake of EPA and DHA increased the circulating amounts of the respective oxylipins in mice (64) and dose-dependently in humans (26). However, this may not apply to ALA in our results, since supplementation with ahiflower oil significantly increased the plasma levels of ALA, without affecting the plasma levels of ALA-derived non-esterified oxylipins. A change in erythrocyte ALA and plasma ALA-derived oxylipins was observed in humans receiving ALA-rich linseed oil in a 12-week intervention (65). However, the ALA dose (12.9 g/d) was much higher than that offered to the participants within the current study (4 g/d). Moreover, in a study by Greupner et al. (65), ALA levels increased after 1 week, while the highest levels of ALA-derived oxylipins were observed after 6 weeks of intervention (65). These results reveal that the oxylipin response was delayed and that the intervention duration of the current study (20 days) was probably too short for ALA oxylipin to be found. In this context, it is worth mentioning a study with obese women, in which the plasma oxylipin pattern was modified by oral supplementation with DHA via fish oil but not ALA-rich flaxseed oil (66). Remarkably, experimental conditions, such as duration of the intervention (28 days vs. 20 days), amount of ALA intake (4 g/day) and sample type (plasma), were similar to those in the current study. In the study by Pauls et al. (66) the plasma levels of EPA and EPA-derived oxylipins were not affected by flaxseed oil intake, which further reveals that EPA-derived oxylipins correspond with plasma EPA levels. In addition, compared to ALA derived from flaxseed oil, SDA derived from ahiflower oil triggers the formation of EPA and EPA-oxylipins more efficiently. It has previously been shown that increasing n3-PUFA status by EPA and DHA supplementation in turn decreases the levels of ARA in erythrocytes and ARA-derived epoxy (CYP450 route) as well as dihydroxy (CYP450/sEH route) oxylipins in human serum (67). Within the current intervention study, ARA and ARA-derived epoxy as well as dihydroxy metabolites were not affected by the ahiflower oil ingestion. Instead, only the levels of 12-HETE and 15-HETE slightly decreased, while EPA counterparts 5-HEPE, 12-HEPE and 15-HEPE increased in response to the ahiflower oil intervention. Furthermore, dihydroxy-EPA metabolites (8,9-DiHETE, 11,12-DiHETE, 14,15-DiHETE, 17,18-DIHETE) were significantly higher following ahiflower oil supplementation, while epoxy-EPA metabolites were not detectable in all plasma samples. Consequently, plant-derived ahiflower oil efficiently increases plasma EPA and corresponding eicosanoids and causes a distinct shift in the entire oxylipin pattern (67).

Fatty acids compete for the enzymes LOX, COX and CYP450. Within the current study cells treated with ALA and SDA contained higher levels of hydroxy ALA, EPA, and DHA metabolites derived from the LOX pathway but hydroxy LA and ARA metabolites were not significantly affected compared to control cells. Moreover, compared to control cells, ALA and SDA supplemented cells contained considerably higher amounts of epoxy-ALA and epoxy-EPA oxylipins. Epoxy-PUFAs can be formed by CYP450 monooxygenases-catalyzed reactions (68). Overall, the present study provides substantial evidence that SDA is a more powerful modulator of EPA and DHA oxylipin levels than ALA. Further investigations are needed to determine if and to what extent SDA acts by enhancing substrate availability (especially EPA) for oxygenation or by stimulating enzyme activity.

In conclusion, ALA and SDA-rich ahiflower oil could serve as an alternative plant-derived source to increase EPA levels in normal weight, healthy humans, while changes in DHA levels are not expected. The increase in plasma EPA levels via ahiflower oil ingestion concomitantly increases EPA-derived hydroxy- and dihydroxy-oxylipins. Additional cell culture results revealed that ALA and SDA increases EPA- levels and EPA-derived oxylipins in HepG2 cells, whereby SDA is more potent than ALA. Taurine does not modulate fatty acid patterns in humans or cultivated HepG2 cells.

## 5 Strengths and limitations of the study

The human intervention study was conducted in a crossover design that accounts for interindividual variability effects. Ahiflower oil and taurine were administered as capsules, which improved the oxidative stability of susceptible n3 fatty acids. Furthermore, the capsules could be stored more easily, daily intake procedures were simpler and the compliance of the participants increased.

The results are limited because the study involved a very homogenous group of participants (young healthy men with normal weight). The duration of the intervention phases was relatively short and could be prolonged to further analyze fatty acids/oxylipins in blood cells (e.g., peripheral blood mononuclear cells, erythrocytes) or clinical biomarkers. The HepG2 cell model was utilized to investigate the impact of supplemented n3-PUFAs on cellular fatty acid profiles, particularly focusing on the primary targets EPA and DHA. Given that isolated fatty acids were provided in high supraphysiological concentrations (50 μmol/L), it cannot be excluded that some fatty acids may remain in the medium, and that cellular uptake and accumulation may vary among fatty acids. Taurine was administered at a concentration of 1 mmol/L, a level considered physiological. Nevertheless, concentration-dependent effects should be anticipated in future investigations.

## Data availability statement

The original contributions presented in the study are included in the article/Supplementary material, further inquiries can be directed to the corresponding author.

## Ethics statement

The studies involving humans were approved by Ethics committee of the medical faculty, Christian-Albrechts-University Kiel, Germany (D 484/18). The studies were conducted in accordance with the local legislation and institutional requirements. The participants provided their written informed consent to participate in this study.

## Author contributions

US: Conceptualization, Data curation, Formal analysis, Investigation, Methodology, Validation, Visualization, Writing – original draft, Writing – review & editing. KE: Data curation, Formal analysis, Investigation, Methodology, Validation, Writing – review & editing. MW: Data curation, Formal analysis, Investigation, Methodology, Validation, Writing – review & editing. KL: Writing – original draft, Writing – review & editing. II: Conceptualization, Data curation, Formal analysis, Validation, Writing – review & editing. FH: Conceptualization, Data curation, Formal analysis, Investigation, Methodology, Validation, Writing – review & editing. PW: Conceptualization, Funding acquisition, Project administration, Supervision, Writing – review & editing. AB-W: Conceptualization, Methodology, Project administration, Supervision, Writing – review & editing. NS: Data curation, Formal analysis, Investigation, Methodology, Validation, Writing – review & editing. GR: Conceptualization, Funding acquisition, Project administration, Resources, Supervision, Validation, Writing – original draft, Writing – review & editing.
